# Chemical Analysis of Extracts from Newfoundland Berries and Potential Neuroprotective Effects

**DOI:** 10.3390/antiox5040036

**Published:** 2016-10-19

**Authors:** Mohammad Z. Hossain, Emily Shea, Mohsen Daneshtalab, John T. Weber

**Affiliations:** School of Pharmacy, Memorial University of Newfoundland, 300 Prince Philip Drive, St. John’s, NL A1B 3V6, Canada; zahidrobin@yahoo.com (M.Z.H.); z25epms@mun.ca (E.S.); mohsen@mun.ca (M.D.)

**Keywords:** anthocyanins, antioxidants, flavonols, *Ribes lacustre*, trauma, *Vaccinium* species

## Abstract

Various species of berries have been reported to contain several polyphenolic compounds, such as anthocyanins and flavonols, which are known to possess high antioxidant activity and may be beneficial for human health. To our knowledge, a thorough chemical analysis of polyphenolics in species of these plants native to Newfoundland, Canada has not been conducted. The primary objective of this study was to determine the polyphenolic compounds present in commercial extracts from Newfoundland berries, which included blueberries (*V. angustifolium*), lingonberries (*V. vitis-idaea*) and black currant (*Ribes lacustre*). Anthocyanin and flavonol glycosides in powdered extracts from *Ribes lacustre* and the *Vaccinium* species were identified using the high performance liquid chromatographic (HPLC) separation method with mass spectrometric (MS) detection. The identified compounds were extracted from dried berries by various solvents via ultrasonication followed by centrifugation. A reverse-phase analytical column was employed to identify the retention time of each chemical component before submission for LC–MS analysis. A total of 21 phenolic compounds were tentatively identified in the three species. Further, we tested the effects of the lingonberry extract for its ability to protect neurons and glia from trauma utilizing an in vitro model of cell injury. Surprisingly, these extracts provided complete protection from cell death in this model. These findings indicate the presence of a wide variety of anthocyanins and flavonols in berries that grow natively in Newfoundland. These powdered extracts maintain these compounds intact despite being processed from berry fruit, indicating their potential use as dietary supplements. In addition, these recent findings and previous data from our lab demonstrate the ability of compounds in berries to protect the nervous system from traumatic insults.

## 1. Introduction

Natural polyphenolic antioxidants have attracted the attention of food scientists because of their positive effects on human health. Many species of berries have high amounts of polyphenolic antioxidants and therefore, are important sources of potential health promoting components [1]. Flavonoids in particular are polyphenols that constitute a large group of secondary plant metabolites [2]. The predominant flavonoids found in berries are anthocyanins and flavonols, which are almost exclusively present in glycosylated forms [3]. The main anthocyanins in fruits are glycosides of six anthocyanidins, with cyanidin as the predominant anthocyanidin, followed by delphinidin, peonidin, pelargonidin, petunidin and malvidin [4,5,6]. Delphinidin is known to be responsible for bluish colours, whereas cyanidin and pelargonidin are responsible for red and purple colours, respectively, in the fruits and vegetables. These compounds have a wide range of biological effects, including potent antioxidant properties, which can protect cells against free radical attack [7,8]. The antioxidant capacity of flavonoids is influenced by the type of sugar moiety, degree of glycosylation, and acylation of anthocyanin glucosides [9,10]. Owing to the large number of flavonoid glycosides present in fruits and the lack of analytical standards available, acid hydrolysis has been used to cleave glycosidic bonds, followed by High Performance Liquid Chromatography (HPLC) in order to quantify the aglycones. A limitation associated with acid hydrolysis of flavonoid glycosides is that the acid concentrations, incubation time and temperature need to be optimised for different classes of flavonoids [11,12]. At present, the most satisfactory method for analysis of mixtures is the multi-step method of separation, isolation and quantification by Liquid Chromatography (LC) with peak identification by Mass spectrophotometry (MS) [3,13]. 

Detailed HPLC analysis has been conducted on several varieties of berries in various areas of Canada [14,15,16], the United States [3,17,18], and Finland [19,20,21,22,23]. The primary objective of this study was to determine the different polyphenolic components present in commercial extracts from *Ribes lacustre* and *Vaccinium* species that grow natively in Newfoundland, with a particular interest in compounds reported to have high antioxidant activity. We also tested the effects of the lingonberry extract for its ability to protect neurons and glia from trauma in vitro.

## 2. Materials and Methods

### 2.1. Samples and Chemicals

Dried powders of *Vaccinium angustifolium* (lowbush blueberry), *Ribes lacustre* (bristly black currant) and *Vaccinium vitis-idaea* (lingonberry; also known as partridgeberry in Newfoundland) were provided from Natural Newfoundland Nutraceuticals (NNN Inc., Markland, NL, Canada). These powders were produced using a Refractance Window^®^ (MCD Technologies, Tacoma, WA, USA) drying technique [24]. The standard cyanidin 3-galactoside was purchased from Chromadex Inc. (Irvine, CA, USA). HPLC grade methanol and acetone were obtained from Caledon laboratories (Georgetown, ON, Canada). Formic acid (HPLC grade) was acquired from Fisher scientific (Fairlawn, NJ, USA).

### 2.2. Extract Preparation

Extracts were prepared similar to the methods of Cho et al. [3]. In brief, samples of berry powders (5 g) were first treated with 20 mL of extract-ion solution containing methanol/water/formic acid (60:37:3 *v*/*v*/*v*). Sonication was performed for 30 min using a Fisher Inc. sonicator (model FS110H) and the mixture was then centrifuged (2614 G) for 30 min, after which the solid was precipitated at the bottom of the tube. Aliquots (4 mL) of supernatant liquid were then subjected to rotary evaporation using a Büchi rotary evaporator at 45 **°**C until at least 95% of extract solvent was removed. The samples were re-suspended in 1 mL of aqueous 3% formic acid solution and then passed through a 0.45 μm nylon filter.

### 2.3. Standard and Calibration Curves

Commercially available anthocyanin reference standard cyanidin 3-galactoside (2.0 g) was dissolved in 10 mL of 3% formic acid to produce concentrations of 200 µg/mL. A serial dilution was conducted in order to obtain concentrations of 150 µg/mL, 100 µg/mL, 50 µg/mL and 25 µg/mL. Standard solutions were injected separately under direct HPLC conditions in order to generate calibration curves for reference compounds. Standard deviation and percentage RSD was also calculated. The % RSD value for all five concentrations was well below 5%. The average values of all five peaks were plotted against the five concentrations and a linear equation was obtained with an R^2^ value of 0.983. 

### 2.4. HPLC Analysis of the Extract Samples

Berry extract samples (15 μL) were analyzed using an Agilent 1100 series HPLC system equipped with a model G1311A Quaternary pump (SL No. DE 40926119). Separation was carried out using a 3.9 mm × 150 mm Symmetry C18 (5 μm) column (SL No. W31761L 008; Agilent technologies, Santa Clara, CA, USA). The system was equilibrated for 20 min at the primary gradient before each injection. Gradients used were the same for all samples with the exception of lingonberry methanol extracts. For most samples the mobile phases used were: (A) 5% formic acid and (B) 100% methanol. Eluent gradient was (a) 0 min—2% B; then gradually (b) 60 min—60% B. For lingonberry methanol extracts the mobile phase was: (A) 0.1% formic acid, and (B) methanol. Eluent gradient was 1% B—0 min-6% 2% B—20 min-12% 3% B—30 min-55%. LC flow rate was one mL/min. Detection of flavonols and anthocyanins was performed with a Diode Array Detector (DAD). We utilized a DAD due to its high specificity and the fact that it has been successfully used to detect the same or similar compounds that we expected to be present in our samples [25,26]. Detection wavelengths for flavonols used were 360 nm and for anthocyanins 520 nm. For our analysis, we utilized similar MS parameters as those developed by Cho et al. [3]. For MS we used the electrospray ionization (ESI) mode. Both positive and negative ionization modes were applied. The parameters were capillary voltage 4.0 kV, nebulizing pressure 30.0 psi, drying gas flow 9.0 mL/min and the temperature was 300 **°**C. Data collection was performed at 1.0 s per cycle with a mass range of *m*/*z* 100—1000. For all extracts, various HPLC chromatograms and their corresponding MS values were compared with values available in the literature for identification purposes.

### 2.5. Cell Culture

Cortical cell cultures were prepared from neonatal rat pups (1–3 days old) according to previously published methods [27,28]. These procedures were approved by the Institutional Animal Care Committee of Memorial University of Newfoundland. Briefly, dissociated cortices were diluted in serum-containing media [Basal Medium Eagles (GIBCO, Grand Island, NY, USA) containing 10% horse serum (GIBCO), an antibiotic-antimycotic solution at a final concentration of 100 units/mL penicillin G, 100 µg/mL streptomycin sulfate, and 250 ng/mL amphotericin B (Sigma, St. Louis, MO, USA), 0.5% glucose (Sigma), 1 mM sodium pyruvate (GIBCO) and 1% N_2_ supplements (GIBCO)] to a concentration of 500,000 cells per mL; cells were then plated in 1-mL aliquots onto collagen-coated six-well FlexPlates (FlexCell, Hillsborough, NC, USA) coated overnight with poly-L-ornithine (500 µg/mL; Sigma). All cultures were maintained in a humidified incubator (5% CO_2_, 37 **°**C). Half of the media in cultures was replaced two days after plating, and then twice per week, with serum-free media containing 2% B27 supplements (GIBCO). Glia formed a confluent monolayer that adhered to the membrane substrate, whereas neurons adhered to the underlying glia. These cultures contained approximately 12% neurons as determined by NeuN, which is a neuronal marker expressed strongly in nuclei and perikarya [29], with the remaining cells representing the glial population. Approximately 95% of NeuN-negative cells stained positively for glial fibrillary acidic protein (Invitrogen, Camarillo, CA, USA), suggesting that the majority of glial cells in these cultures were composed of astrocytes. These cultures were used for experiments at 9–15 days in vitro.

### 2.6. Cell Injury

Cortical cultures were stretch-injured using a model 94 A Cell Injury Controller developed by Ellis et al. [30] (Bioengineering Facility, Virginia Commonwealth University, Richmond, VA, USA). In brief, the Silastic membrane of the FlexPlate well is rapidly and transiently deformed by a 50-ms pulse of compressed nitrogen or air, which deforms the Silastic membrane and adherent cells to varying degrees controlled by pulse pressure. The extent of cell injury—produced by deforming the Silastic membrane on which the cells are grown—is dependent on the degree of deformation, or stretch. Based on previous work, we used a level of cell stretch equivalent to 5.5 mm deformation (31% stretch), which has been defined as a “mild” level of injury [30,31]. Lingonberry extract (1 μL) was added to the cell cultures (in 1 mL of media) 15 min before injury. One μL of solvent (3% formic acid) was added to some cultures to assess the effect of the solvent alone on cells. 

### 2.7. Cell Counts and Statistical Analysis

Cell cultures were fixed for 20 min with 4% paraformaldehyde as previously described [25,26]. Cultures were then dehydrated with ethanol and mounted with 4′,6-diamidino-2-phenylindole (DAPI), which labels the nuclei of all cells. Images were captured using a Zeiss ObserverA1 microscope and a Pixelfly qe CCD camera (pco., Kelheim, Germany) using AxioVision software (Zeiss). DAPI-positive cells were counted in five separate fields in each culture well at a magnification of 40×. The data for the bioactivity (cell culture) experiments were analyzed with one-way ANOVA (*p* < 0.05) followed by Tukey’s multiple comparisons test, and are expressed as % of control values.

## 3. Results

### 3.1. Analysis of Lingonberry (V. vitis-idaea) Extracts

Lingonberry samples were treated with extraction solvent [MeOH/H_2_O/HCOOH (60:37:3 *v*/*v*/*v*)], processed and analyzed by HPLC–MS as described in the materials and methods section. Chemical compounds were identified from the HPLC peaks and are summarized in Table 1. We have used the method described by Cho et al. [3] for extraction of various berry samples as described in the materials and methods section. However, this method did not give satisfactory extraction for all samples. Therefore, for the analysis of some of the lingonberry extracts, the sample was first treated with acidified methanol [MeOH in 1M HCl (85:15 *v*/*v*)]. During the analysis the sample: solvent ratio was 1:8. The pH was adjusted to 1.0 (25 °C) using 1M HCl. We used the methodology similar to that developed by Hosseinian and Beta [15]. The sample solvent mixture was sonicated for 30 min and the mixture was transferred to a 50 mL Falcon tube, which was centrifuged at 2614 G for 45 min. Supernatant was collected and subjected to rotary evaporation until 95% of its original volume was removed, which was then spun at 2614 G for another 20 min. The supernatant obtained was filtered using a 0.45 µm nylon filter prior to analysis using the LC–MS instrument. Different compounds using this procedure were identified from the HPLC peaks and are summarized in Table 2. The two different solvent systems were used in order to determine different compounds present in the lingonberry extracts. It was assumed that some compounds which were not extracted in the first solvent system used (MeOH/H_2_O/HCOOH) may be isolated in the second solvent system (MeOH/HCl) and more easily detected. This approach isolated additional compounds such as proanthocyanidin A, quercetin-3-glucoside and quercetin-3-O-α arabinoside, which were not detected in the first solvent system.

### 3.2. Confirmation and Quantification of Cyanidin-3-Galactoside in Lingonberry Extracts

We chose to quantify cyanidin-3-galactoside in *V. vitis-idaea* as this compound has been reported to be the major anthocyanin in this species [32]. It was found that the retention time (17.70 min) for LC peaks of cyanidin-3-galactoside present in *V. vitis-idaea* extracts was similar to the retention time for LC peaks of the cyanidin-3-galactoside standard, which confirms the presence of this compound in the extracts. From HPLC analysis the area under peak was found to be 3978.62. From the area vs. concentration graph after calculation the concentration of cyanidin-3-galactoside was obtained as 133.71 µg/mL. Therefore, in 100 g of extract the amount of cyanidin-3-galactoside was 66.33 mg or 0.06633 g. Therefore, the concentration of cyanidin-3-galactoside in the *V. vitis-idaea* sample was 0.066%.

### 3.3. Analysis of Blueberry (V. angustifolium) Extracts

The blueberry extracts were first treated with 40 mL of 2M HCl in ethanol. Hydrolysis was performed at 90 °C in an oil bath for 90 min to break the glycoside linkage. We used a method similar to Nyman and Kumpulainen [20]. Hydrolysis was conducted to produce the flavylium ion (aglycon part), which was identified in the MS system. After hydrolysis, the extract solution was diluted and filtered using a 0.45 µm nylon filter. The sample was then analyzed using a LC–MS instrument. Conditions for MS were the same as those of other analyses. Different compounds identified in blueberry ethanol extracts are summarized in Table 3. The HPLC peaks were identified based on comparison of detected and calculated molecular ions as mentioned in the literature [3,15]. It is to be noted that there is a lack of reproducibility in exact retention times as these may be influenced by aspects such as room temperature, the column and the solvent system. We found very similar retention times when using the same solvent however, so we strongly believe that the different retention times were due to the various solvents.

In a separate experiment, blueberry extracts were first treated with acidified methanol (20 mL) [MeOH in 1M HCl (85:15 *v*/*v*)]. In this experiment the method used was similar to that of Hosseinian and Beta [15]. This experiment was conducted in order to evaluate whether any additional compounds would be detected using acidified methanol rather than acidified ethanol as the solvent due to polarity. Acidified methanol also acts as a very good solvent system for anthocyanin compounds. Various chemical compounds were identified from the LC peaks and are summarized in Table 4. 

### 3.4. Analysis of Black Currant (R. lacustre) Extracts

For the analysis of the black currant extracts, the samples were first treated with acidified methanol. The pH was adjusted to 1.0 (25 °C) and 1M HCl was used for this purpose. Various compounds identified in *R. lacustre* are summarized in Table 5. In a separate experiment the black currant sample was treated with 70% aqueous acetone having 0.01M HCl (20.0 mL) and sonicated for 20 min, as reported by Anttonen and Karjalainen [21]. The compounds identified in black currant extracts by this method are listed in Table 6. The HPLC peaks were identified based on comparison of detected and calculated molecular ions as mentioned in previous reports [3,21]. 

### 3.5. Protective Effect of Lingonberry Extract Against Traumatic Injury

**Figure 1:**
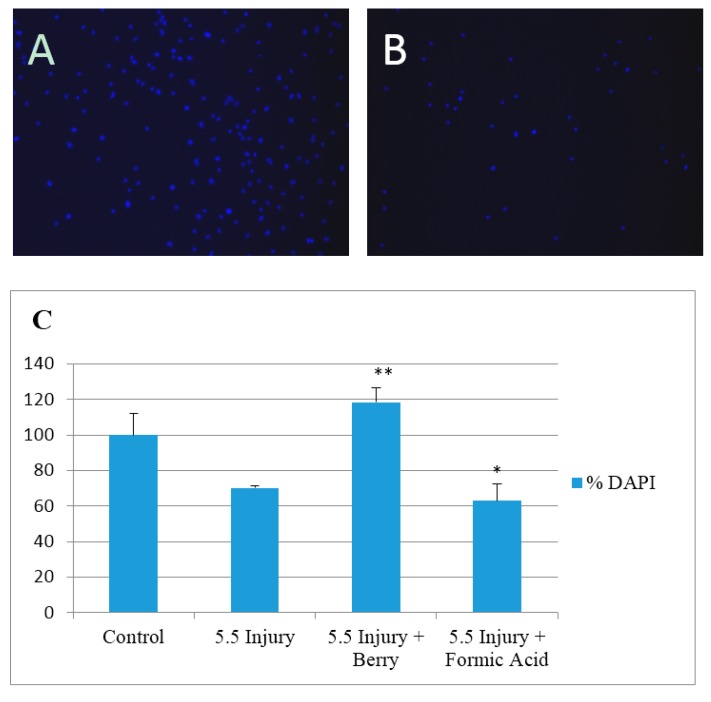
Protective effects of lingonberry extract against in vitro trauma. (**A**) Representative image of DAPI stained nuclei (**blue**) in an uninjured cortical cell well at 9 days in vitro (DIV); (**B**) Representative image of DAPI stained nuclei (**blue**) in an injured cortical cell well (5.5 mm injury) at 9 DIV; (**C**) % of control of DAPI stained nuclei of cortical cells 9–15 DIV under various conditions (*n* = 6 wells in each condition from 3 different culture preparations). Formic acid refers to solvent alone. * statistically different from control group *p* < 0.05. ** Statistically different from 5.5 mm injury group *p* < 0.01. Values are mean ± S.E.M.

In preliminary studies we found that treatment of cultures with 1 µL of solvent alone had no significant change in cell number after 24 h. Injury caused ~30% cell loss by 24 h after injury (Figure 1). Injury in the presence of solvent alone caused a similar loss of cells while the effect of injury was completely reversed in presence of the lingonberry fruit extract (added 15 min before injury). This suggests a high protective effect of lingonberry fruit against traumatic injury in rat brain cultures. 

## 4. Discussion

### 4.1. Comparisons to Findings from Various Berry Species in Other Regions

In our lingonberry extracts, only six compounds were identified (See Table 7). A previous report conducted on *V. vitis-idaea* growing in Finland describes the identification of as many as 28 compounds in methanolic extracts of that species [22]. Comparing HPLC conditions we found that this report used similar analytical conditions as used by our lab. We are unsure of the explanation for this difference in the number of compounds, considering that we obtained excellent peak separation during analysis of the extract and the drying method used to produce the original extract maintains at least 94% (label claimed) of the bioactive compounds intact. It is well known that the chemical composition differs significantly depending on soil, subspecies and climates. As these berries were grown in Newfoundland compared to Finland, it is possible that due to different conditions the number of compounds may vary. We tested levels of cyanidin-3-galactoside in extract samples from *V. vitis-idaea*. We successfully detected a level of 66.33 mg/100 g cyanidin-3-galactoside in Newfoundland *V. vitis-idaea*, which is noteworthy since this compound is considered to have fairly high antioxidant activity [33]. 

In blueberry powder, a total of 11 compounds were identified (Table 7). Among them, 9 compounds were anthocyanins and two were flavonols. Cho et al. [3] reported that blueberry genotypes including two commercial cultivars found in the United States, Bluecrop (*V. corymbosum* L) known as Northern Highbush and Ozarkblue (a hybrid of majority *V. corymbosum* with some contribution from *V. darrowi* L and *V. ashei*), also known as Southern Highbush, contained 17 and 15 anthocyanins, respectively. This suggests that more compounds may be present in blueberry species found in the United States compared to those of Newfoundland, which may be due to climate, soil and collection time, as the extraction method and analysis conditions were the same. However, our blueberry samples are from a different species (*V. angustifolium*) as compared to the blueberry cultivars analyzed by Cho et al. [3]. Therefore, blueberry cultivars may contain more anthocyanins than naturally growing *V. angustifolium* in Newfoundland. The amount of different anthocyanins detected in our extract powder sample was less than previously reported for *V. angustifolium* and *V. myrtilloides* wild blueberries found in Quebec [16] and Manitoba [15]. This may be due to the fact that those studies were conducted in berry fruits versus extract powders, or to differences in the natural composition of the berries.

In black currant six compounds were identified including chlorogenic acid (3-caffeoylquinic acid), myrecitin-3-rhamnoside, quercetin-3-rutinoside, quercetin glucoside kaempferol rutinoside and kaempferol glucoside (Table 7). This represents a different profile of compounds compared to previous work conducted on black currant (*R. nigrum*) species growing in Finland [20,21] as well as black currant cultivars in Finland [19]. Slimestad and Solheim [34] worked on *R. nigrum* available in Norway and reported that 15 anthocyanins were present in those berries. Different extraction processes and HPLC conditions may be the reason that the number of compounds identified in *R. nigrum* found in Norway was more than in our findings in *R. lacustre*. Then again, the chemical profiles may represent a distinct difference between *R. nigrum* and *R. lacustre*. Määttä et al. [35] identified eight anthocyanins in acidified methanolic extracts of *R. nigrum* available in Sweden. Mikkonen et al. [19] identified four compounds, myricetin, morin, quercetin and kaempferol, in black currant cultivars grown in Finland, which show similarity to our findings. 

### 4.2. Potential Bioactivities of the Analyzed Berry Species

Antioxidant compounds obtained in the diet or through dietary supplementation could be beneficial for combating reactive oxygen and nitrogen species, which can contribute to the development of several disorders, including cancer, cardiovascular disease, diabetes and neurodegeneration [33,36,37]. Our laboratory has a specific interest in identifying natural compounds that may be neuroprotective. We feel that berries or their constituents could potentially be useful for treating or preventing rapid brain aging [38], and may even be useful for decreasing damage associated with more severe ailments such as stroke and traumatic brain injury [39]. However, the extent to which polyphenols can cross the blood brain barrier is largely unknown [40]. Recently, we have found that cortical cells derived from rat brains are protected from glutamate-mediated neurotoxicity by extracts derived from locally collected Newfoundland *V. angustifolium* and *V. vitis-idaea* fruits and leaves [28]. The extracts from blueberry fruits were further tested using the in vitro trauma model described in this paper, and they were found to be highly protective against injury (unpublished data). Interestingly, we found that the leaves of these two species had a higher polyphenolic content and free radical scavenging ability as compared to the fruits. Other studies have also found that the leaves of *V. angustifolium* and *V. vitis-idaea* have a high content of polyphenolic compounds [14,41] suggesting potential sources of dietary supplements. 

## 5. Conclusions

Overall, the results suggest that various species of berries growing natively in Newfoundland contain a wide array of anthocyanins and flavonols with high levels of antioxidant capacity. Further investigation is required to confirm other polyphenols potentially present in these species and to quantify levels of various compounds with antioxidant capacity. In addition, this data demonstrates that the drying technique used to produce these extracts maintains several polyphenolic compounds in the samples, suggesting that these forms of powdered berry extracts could be viable dietary supplements. Further experimentation is aimed at conducting detailed chemical analyses of the leaves of these species, deciphering which species of berries are most neuroprotective, and determining if specific polyphenols are responsible for neuroprotection. In addition, the extent to which polyphenols cross the blood brain barrier is currently being investigated in rodent models.

## Figures and Tables

**Table 1:** Compounds identified in lingonberry (*V. vitis-idaea*) MeOH/H_2_O/HCOOH extracts.

No.	HPLC RT (min)	Identification	*m*/*z* Values		*m*/*z* Values in Literature ^1^
			[M−H]^−^	[M+H]^+^	[M+H]^+^
1	16.18	Cyanidin-3-glucoside	447.1	449.1	449
2	17.70	Cyanidin-3-galactoside		449.1	449
3	18.54	Cyanidin-3-arabinoside		419.1	419

There were some other unknown peaks in the HPLC chromatogram, which could not be identified. ^1^ Ek et al., (2006) [22].

**Table 2:** Compounds identified in lingonberry (*V. vitis-idaea*) methanol extracts.

No.	HPLC RT (min)	Identification	*m*/*z* Value	*m*/*z* Value in Literature ^1^
		Anthocyanins	[M+H]^+^	[M+H]^+^
1	25.62	Cyanidin-3-glucoside	449.2	449
2	25.92	Cyanidin-3-galactoside	449.1	449
3	27.49	Proanthocyanidin A	577.1	577
4	31.34	Quercetin-3-glucoside	465.1	465
5	32.28	Quercetin-3-O-α arabinoside	435.1	435

There were some other unknown peaks in the HPLC chromatogram, which could not be identified. ^1^ Ek et al., (2006) [22].

**Table 3:** Compounds identified in blueberry (*V. angustifolium*) ethanol extracts.

No.	HPLC RT(min)	Identification	*m*/*z* Value	*m*/*z* Value in Literature ^1,2^
		Anthocyanins	[M]^+^	[M]^+^
1	10.08	Delphinidin-3-galactoside	465.1	465
2	10.52	Delphinidin-3-glucoside	465.1	465
3	10.88	Cyanidin-3-galactoside	449.1	449
4	11.19	Delphinidin-3-arabinoside	435.1	435
5	12.19	Petunidin-3-glucoside	479.1	479
6	13.48	Malvidin-3-glucoside	493.1	493
7	14.14	Peonidin-3-glucoside	463.1	463

There were some other unknown peaks in the HPLC chromatogram, which could not be identified. ^1^ Cho et al., (2004) [3]; ^2^ Hosseinian and Beta (2007) [15].

**Table 4:** Compounds identified in blueberry (*V. angustifolium*) methanol extracts.

No.	HPLC RT(min)	Identification	*m*/*z* Value	*m*/*z* Value in Literature ^1,2^
		Anthocyanins	[M]^+^	[M]^+^
1	22.68	Delphinidin-3-galactoside	465.1	465
2	23.71	Delphinidin-3-arabinoside	435.1	435
3	24.70	Cyanidin-3-galactoside	449.1	449
4	25.90	Petunidin-3-galactoside	479.1	479
5	30.24	Malvidin-3-galactoside	493.1	493
6	33.15	Peonidin-3-glucoside	463.1	463
		**Flavonols**	**[M]^−^**	**[M]^−^**
7	20.50	Myricetin-3-rhamnoside	463.0	463
8	22.20	Quercetin-3-galactoside	463.0	463

There were some other unknown peaks in the HPLC chromatogram, which could not be identified. ^1^ Cho et al., (2004) [3], ^2^ Hosseinian and Beta (2007) [15].

**Table 5:** Compounds identified in black currant (*R. lacustre*) methanol extracts.

No.	HPLC RT(min)	Identification	*m*/*z* Values [M]^+^ or [M+H]^+^	*m*/*z* Values in Literature^1,2^ [M]^−^ or [M−H]^−^
1	1.55	chlorogenic acid	353.0	353
2	10.48	myricetin-3-rhamnoside	463.1	463
3	11.05	quercetin-3-rutinoside	609.2	609
4	12.03	kaempferol rutinoside	595.2	595

There were some other unknown peaks in the HPLC chromatogram, which could not be identified. ^1^ Anttonen and Karjalainen (2006) [21]; ^2^ Cho et al., (2004) [3].

**Table 6:** Compounds detected in black currant (*R. lacustre*) acetone extracts.

No.	HPLC RT(min)	Identification	*m*/*z* Values [M]^+^ or [M+H]^+^	*m*/*z* Values in Literature ^1,2^ [M]^−^ or [M−H]^−^
1	1.45	chlorogenic acid	353.0	353
2	7.45	quercetin glucoside	465.1	465
3	8.57	kaempferol glucoside	449.1	449

There were some other unknown peaks in the HPLC chromatogram which could not be identified. ^1^ Anttonen and Karjalainen (2006) [21]; ^2^ Cho et al., (2004) [3].

**Table 7:** Summary of compounds present in the various berry extracts.

Compounds	Blueberry *V. angustifolium*	Black Currant *R. lacustre*	Lingonberry *V. vitis-idaea*
Delphinidin-3-galactoside	X		
Delphinidin-3-glucoside	X		
Delphinidin-3-arabinoside	X		
Cyanidin-3-galactoside	X		X
Cyanidin-3-glucoside			X
Cyanidin-3-arabinoside			X
Petunidin-3-galactoside	X		
Petunidin-3-glucoside	X		
Peonidin-3-glucoside	X		
Malvidin-3-galactoside	X		
Malvidin-3-glucoside	X		
Chlorogenic acid		X	
Myrecitin-3-rhamnoside	X	X	
Quercetin-3-galactoside	X		
Quercetin-3-rutinoside		X	
Kaempferol rutinoside		X	
Quercetin glucoside		X	
Kaempferol glucoside		X	
Proanthocyanidin A			X
Quercetin-3-glucoside			X
Quercetin-3-O-α arabinoside			X
